# Standardised inventories of spiders (Arachnida, Araneae) of Macaronesia II: The native forests and dry habitats of Madeira archipelago (Madeira and Porto Santo islands)

**DOI:** 10.3897/BDJ.8.e47502

**Published:** 2020-01-14

**Authors:** Jagoba Malumbres-Olarte, Mário Boieiro, Pedro Cardoso, Rui Carvalho, Luís Carlos Fonseca Crespo, Rosalina Gabriel, Nuria Macías Hernández, Octávio S. Paulo, Fernando Pereira, Carla Rego, Alejandra Ros-Prieto, Isamberto Silva, Ana Vieira, François Rigal, Paulo A. V. Borges

**Affiliations:** 1 CE3C – Centre for Ecology, Evolution and Environmental Changes / Azorean Biodiversity Group and Universidade dos Açores, Angra do Heroísmo, Azores, Portugal CE3C – Centre for Ecology, Evolution and Environmental Changes / Azorean Biodiversity Group and Universidade dos Açores Angra do Heroísmo, Azores Portugal; 2 LIBRe – Laboratory for Integrative Biodiversity Research, Finnish Museum of Natural History, University of Helsinki, Helsinki, Finland LIBRe – Laboratory for Integrative Biodiversity Research, Finnish Museum of Natural History, University of Helsinki Helsinki Finland; 3 IUCN SSC Spider & Scorpion Specialist Group, Helsinki, Finland IUCN SSC Spider & Scorpion Specialist Group Helsinki Finland; 4 Biodiversity Research Institute UB, Departament Department of Evolutionary Biology, Ecology and Environmental Sciences (Arthropods), Barcelona, Spain Biodiversity Research Institute UB, Departament Department of Evolutionary Biology, Ecology and Environmental Sciences (Arthropods) Barcelona Spain; 5 Island Ecology and Evolution Research Group, IPNA-CSIC, Tenerife, Canary Islands, Spain Island Ecology and Evolution Research Group, IPNA-CSIC Tenerife, Canary Islands Spain; 6 Faculdade de Ciências da Universidade de Lisboa, Departamento de Biologia Animal e Centro de Biologia Ambiental, Computational Biology and Population Genomics Group, Lisbon, Portugal Faculdade de Ciências da Universidade de Lisboa, Departamento de Biologia Animal e Centro de Biologia Ambiental, Computational Biology and Population Genomics Group Lisbon Portugal; 7 Instituto das Florestas e da Conservação da Natureza, Funchal, Portugal Instituto das Florestas e da Conservação da Natureza Funchal Portugal; 8 Environment and Microbiology Team, Université de Pau et des Pays de l'Adour, Pau Cedex, France Environment and Microbiology Team, Université de Pau et des Pays de l'Adour Pau Cedex France; 9 IUCN - SSC Mid-Atlantic Island Invertebrates Specialist Group, Angra do Heroísmo, Azores, Portugal IUCN - SSC Mid-Atlantic Island Invertebrates Specialist Group Angra do Heroísmo, Azores Portugal

**Keywords:** Arthropoda, Araneae, Madeira, Porto Santo, native forest, dry habitat, exotic species, standardised sampling

## Abstract

**Background:**

Here we present the data obtained from the samples collected as part of a large research project (MACDIV) which aims at understanding the drivers of spider (Araneae) community assembly in Macaronesian islands. To obtain the data, we applied the sampling protocol COBRA (Conservation Oriented Biodiversity Rapid Assessment), in twelve 50 m x 50 m native forest plots and five dry habitat plots on the island of Madeiraand in 5 dry habitat plots on the island of Porto Santo. Through this publication, we contribute to the knowledge of the arachnofauna of the Madeiran archipelago.

**New information:**

From the samples that we collected, we obtained a total of 14,902 specimens, of which 49% were adults (7,263). We identified these specimens to 87 species and 18 morphospecies (undescribed), belonging to 26 families. Species of the family Linyphiidae dominated the samples, with 24 (morpho)species. Out of the 105 recorded (morpho)species, 34 were endemic, 26 native non-endemic, 22 introduced and 23 species of unknown origin. We report seven new records of possibly recently introduced species in the Madeiran archipelago. We also present 21 new records for Madeira island and 32 for Porto Santo (33 for the whole archipelago).

## Introduction

The north Atlantic archipelago of Madeira is composed of the volcanic islands of Madeira, Porto Santo and the Desertas, as well several islets. At approximately 700 km from the African coast and at more than 400 km from the Canary Islands, it is the second most isolated Macaronesian archipelago, after the Azores. This intermediate distance, combined with an geological age of 5-27 My, has allowed frequent colonisation rates and enough diversification time to generate diverse species communities (Fernández-Palacios 2010, Fernández-Palacios et al. 2011). Since its settlement in the XV Century by the Portuguese, the archipelago has gone through a profound environmental transformation. Nevertheless, the main island of the archipelago, i.e. Madeira, still preserves a considerable area of unique native laurel forest (laurisilva), covering 20% of the island (Boieiro et al. 2015, Boieiro et al. 2018).

The laurisilva is indeed a unique and iconic ecosystem, that is often seen as representative of the Macaronesian archipelagos as a whole and contains a great deal of endemic species (Boieiro et al. 2010, Boieiro et al. 2013, Borges et al. 2008). However, the archipelago is also the home of more open habitats, such as scrublands and thermophilous grasslands, present in drier conditions than those where laurisilva is found. Although these often overlooked dry habitats may not be as species-rich as forests, they do contribute to the endemic fauna of the archipelago, which represent 20% of all the species (Borges et al. 2008). Moreover, Madeiran dry habitats may also contain a substantial number of unknown species that have eluded recent taxonomic, conservation and faunistic research, including IUCN Red List assessments (Martín et al. 2008, Martín et al. 2010, Silva et al. 2008, Cardoso et al. 2017), as well as unrecorded exotic species (Silva et al. 2008).

The need for an update in many taxa is exemplified by spiders. Although, according to the last available checklist (Cardoso and Crespo 2008), the spider fauna of Madeira archipelago is composed of 183 species (including 58 endemic species), new taxa have been discovered since then (e.g. Crespo et al. 2009, Crespo et al. 2014).

This publication is the second of a series on Macaronesian spider fauna (see Malumbres-Olarte et al. 2019) and provides habitat, biogeographic and colonisation information on the species collected in 12 native forest plots and 10 native dry habitat plots on the islands of Madeira and Porto Santo through the project MACDIV.

## Sampling methods

### Study extent

**Figure 2.**
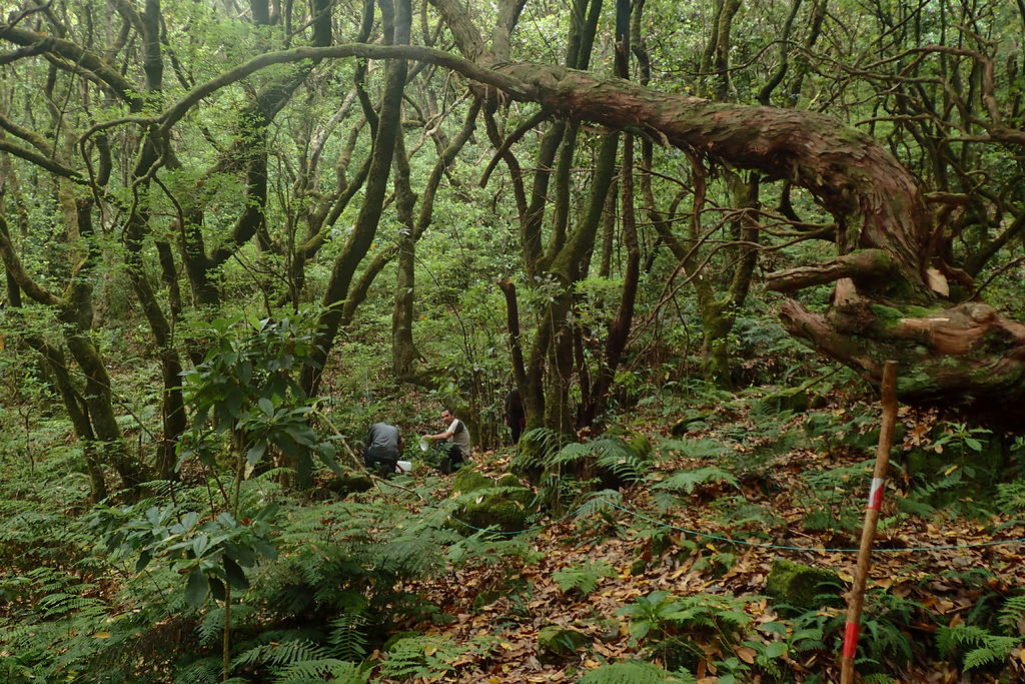
Forest habitat Plot 3 of Madeira island (Credit: Jagoba Malumbres-Olarte).

**Figure 3.**
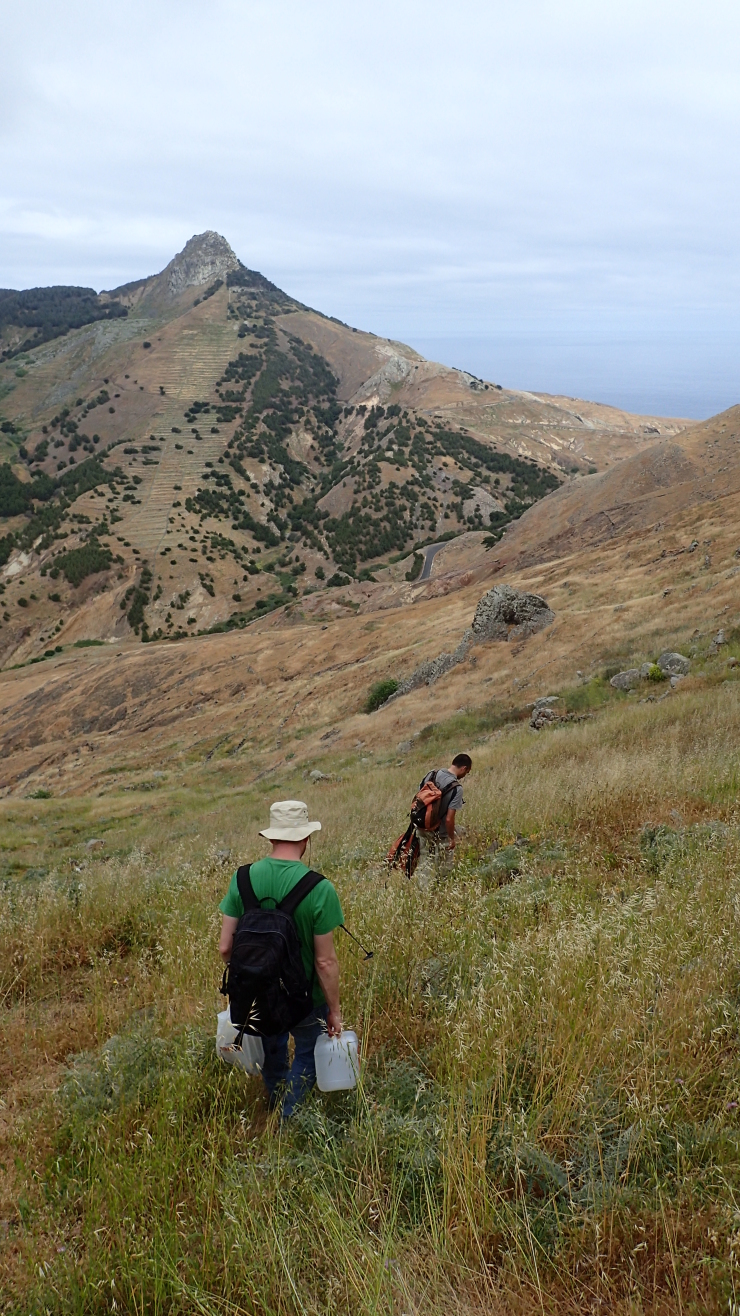
Dry habitat Plot 1 of the island of Porto Santo (Credit: Jagoba Malumbres-Olarte).

We established twenty-two 50 m × 50 m plots, grouped according to habitat and between-plot distances. Twelve plots were located in areas covered with laurisilva and grouped in two sets of six. Within each set, plots were placed at increasing distances from a first, reference plot (Table 1): 0.1, 1, 5, 10 and 20 km (Fig. 1). The remaining 10 plots were located in areas of open dry habitat, five on Madeira island and five on Porto Santo, also at increasing distances from a reference plot (0.1, 1, 5 and 10 km). This design allowed for testing of distance decay patterns on beta diversity on a log scale. We set up the forest plots in well-preserved native forest areas, where native tree species, such as *Clethra arborea*, *Laurus novocanariensis*, *Ocotea foetens* and *Persea indica*, were dominant (Neves et al. 1996, Menezes et al. 2005) (Fig. 2). We set the dry plots in grasslands at low-altitude, where the vegetation cover was dominated by herbaceous species and several shrubs, like *Echium* spp, *Euphorbia piscatoria* and *Globularia salicina* (Medeiros et al. 2010) (Fig. 3).

### Sampling description

We applied two versions of the optimised and standardised COBRA protocol (Conservation Oriented Biodiversity Rapid Assessment) (Cardoso 2009): the protocol for temperate forests (which we applied in forest plots) and the protocol for open habitats (applied to dry habitat plots). The COBRA protocols have been proposed as part of standard inventorying and monitoring programmes on island and continental ecosystems and have already been used for a number of studies on spiders and beetles (Cardoso 2009, Borges et al. 2018, Malumbres-Olarte et al. 2017, Malumbres-Olarte et al. 2018, Crespo et al. 2018). The forest COBRA protocol consisted of: four night aerial samples (1 hour / sample), two day sweeping samples and two night sweeping samples (1 hour / sample), two day beating samples and two night beating samples (1 hour / sample) and 12 pitfall samples (4 traps / sample). In addition, we collected the following samples to also cover beetle diversity (beetle data will be included in future publications): two diurnal active aerial searching under bark, lichens and bryophytes (ABS) (1 hour / sample) and two diurnal active aerial searching in decaying trunks, dead wood on the ground and under stones (GWS) (1 hour / sample). The protocol for dry open areas was composed of: four night ground samples (1 hour / sample) and four day sweeping samples and four night sweeping samples (1 hour / sample). Sampling occurred in August 2016 (forest habitat plots of Madeira) and April 2017 (dry habitat plots of Madeira and Porto Santo).

## Geographic coverage

### Description

Madeira and Porto Santo islands, Madeira, Macaronesia, Portugal

### Coordinates

32.66138 and 33.0921 Latitude; -17.15871 and -16.30453 Longitude.

## Taxonomic coverage

### Taxa included

**Table:** 

Rank	Scientific Name	Common Name
order	Araneae	Spiders

## Temporal coverage

**Data range:** 2016-8-01 – 2017-4-30.

### Notes

Sampling in the native forest occurred in August 2016. Sampling in dry habitats occurred in April 2017.

## Collection data

### Collection name

Dalberto Teixeira Pombo insect collection at the University of Azores

### Collection identifier

DTP

### Specimen preservation method

All specimens were preserved in 96% ethanol

### Curatorial unit

Dalberto Teixeira Pombo insect collection at the University of Azores (Curator: Paulo A. V. Borges)

## Usage rights

### Use license

Open Data Commons Attribution License

## Data resources

### Data package title

MACDIV_COBRA_Madeira_Forest_and_Dry

### Resource link

http://ipt.gbif.pt/ipt/resource?r=spiders_madeira

### Alternative identifiers

http://islandlab.uac.pt/software/ver.php?id=38

### Number of data sets

1

### Data set 1.

#### Data set name

MACDIV_COBRA_Madeira_Forest_and_Dry

#### Data format

Darwin Core Archive

#### Number of columns

62

#### Download URL

http://ipt.gbif.pt/ipt/resource?r=spiders_madeira

#### Data format version

version 1

#### Description

The following data table includes all the records for which a taxonomic identification of the species was possible. The dataset submitted to GBIF is structured as a sample event dataset, with two tables: event (as core) and occurrences. The data in this sampling event resource have been published as a Darwin Core Archive (DwCA), which is a standardised format for sharing biodiversity data as a set of one or more data tables. The core data table contains 562 records (eventID). One extension data table also exists with 3281 occurrences. An extension record supplies extra information about a core record. The number of records in each extension data table is illustrated in the IPT link. This IPT archives the data and thus serves as the data repository. The data and resource metadata are available for downloading in the downloads section.

**Data set 1.** 

Column label	Column description
Table of Sampling Events	Table with sampling events data (beginning of table)
id	Unique identification code for sampling event data
eventID	Identifier of the events, unique for the dataset
samplingProtocol	The sampling protocol used to capture the species
sampleSizeValue	The numeric amount of time spent in each sampling
sampleSizeUnit	The unit of the sample size value
samplingEffort	The amount of time of each sampling
eventDate	Date or date range the record was collected
eventTime	Time of the day the record was collected
startDayOfYear	The earliest ordinal day of the year on which the event occurred
endDayOfYear	The latest ordinal day of the year on which the event occurred
year	Year of the event
month	Month of the event
day	Day of the event
habitat	The surveyed habitat
fieldNumber	The code given to each sample
locationID	Identifier of the location
islandGroup	Name of archipelago
island	Name of the island
country	Country of the sampling site
countryCode	ISO code of the country of the sampling site
stateProvince	Name of the region of the sampling site
locationRemarks	Details on the locality site
decimalLatitude	Approximate centre point decimal latitude of the field site in GPS coordinates
decimalLongitude	Details on the locality site
Details on the locality site	The reference point for the various coordinate systems used in mapping the earth
coordinateUncertaintyInMetres	Uncertainty of the coordinates of the centre of the sampling plot
coordinatePrecision	Precision of the coordinates
georeferenceSources	A list (concatenated and separated) of maps, gazetteers or other resources used to georeference the Location, described specifically enough to allow anyone in the future to use the same resources.
Table of Species Occurrence	Table with species abundance data (beginning of new table)
id	Unique identification code for species abundance data
type	Type of the record, as defined by the Public Core standard
licence	Reference to the licence under which the record is published
institutionID	The identity of the institution publishing the data
collectionID	The identity of the collection publishing the data
institutionCode	The code of the institution publishing the data
collectionCode	The code of the collection where the specimens are conserved
datasetName	Name of the dataset
basisOfRecord	The nature of the data record
dynamicProperties	The name of the scientific project funding the sampling
occurrenceID	Identifier of the record, coded as a global unique identifier
catalogNumber	Record number of the specimen in the collection
recordedBy	Name of the person who performed the sampling of the specimens
individualCount	Total number of individuals captured
organismQuantityType	The unit of the identification of the organisms
sex	The sex and quantity of the individuals captured
lifeStage	The life stage of the organisms captured
establishmentMeans	The process of establishment of the species in the location, using a controlled vocabulary: 'naturalised', 'introduced', 'endemic', "unknown"
occurrenceStatus	Information about the presence/absence of the species
eventID	A unique identifier of an occurrence
identifiedBy	Name of the person who made the identification
dateIdentified	Date on which the record was identified
scientificName	Complete scientific name including author and year
kingdom	Kingdom name
phylum	Phylum name
class	Class name
order	Order name
family	Family name
genus	Genus name
specificEpithet	Specific epithet
taxonRank	Lowest taxonomic rank of the record
scientificNameAuthorship	Name of the author of the lowest taxon rank included in the record

## Additional information

### Results

We collected a total of 14,902 specimens – of which 49% were adults (7,263) - belonging to 105 (morpho) species and 26 families (Tables 2, 3, 4) - 87 species and 18 morphospecies (undescribed) (Malumbres-Olarte et al. 2019b). The number of species per plot oscillated between 19-32, with the minimum in the dry Plot 4 of Porto Santo (PS4) and the maximum number in Plot 3 of Madeiran forest (MF3). Out of the recorded (morpho)species, 34 were endemic, 26 native non-endemic, 22 introduced and 23 species of unknown origin. We report seven new records of introduced species in the Madeiran archipelago. On Madeira island, we recorded 88 (morpho)species, of which 26 were endemic, 26 native non-endemic, 19 introduced and 17 species of unknown origin. On Porto Santo island, we recorded 48 (morpho)species, of which 12 were endemic species, 7 native non-endemic species, 14 introduced species and 15 species of unknown origin. We present 21 new records for Madeira island and 32 for Porto Santo (33 for the whole archipelago).

The most widespread (morpho)species were *Tenuiphantes tenuis* (Blackwall, 1852) (introduced), *Porrhoclubiona decora* (Blackwall, 1859) (native non-endemic), *Neoscona crucifera* (Lucas, 1838) (introduced), *Microlinyphia johnsoni* (Blackwall, 1859) (native non-endemic), *Cryptachaea blattea* (Urquhart, 1886) (introduced), *Cheiracanthium albidulum* (Blackwall, 1859) (endemic), *Ceratinopsis acripes* (Denis, 1962) (native non-endemic), *Meta stridulans* Wunderlich, 1987 (endemic), *Echinotheridion gibberosum* (Kulczyński, 1899) (native non-endemic), *Episinus maderianus* Kulczyński, 1905, (native non-endemic) and *Paidiscura orotavensis* (Schmidt, 1968) (native non-endemic). The five most abundant species were *Zodarion styliferum* (Simon, 1870) (11%, unknown origin), *Episinus maderianus* Kulczyński, 1905 (9%, native non-endemic), *Diplocephalus graecus* (O.P.-Cambridge, 1873) (8%, introduced), *Tenuiphantes tenuis* (Blackwall, 1852) (7%, introduced) and *Macaridion barreti* (Kulczyński, 1899) (7%, native non-endemic).

### Taxonomic and biogeographic classification

In this section, we point out, explain and discuss the identification of some of the species and morphospecies found – the ones for which information is limited and ordered as in Tables 2, 3 and 4 – and their classification as *endemic, native non-endemic, introduced* and *unknown*.

*Synaphris saphrynis*

This species is known from Spain and Selvagens Islands. It is also present on Bugio island (Desertas), as one of the authors (L. Crespo) identified a specimen collected by M. Boieiro et al. in 2011/12 (unpublished record). The limited number of available records does not allow us to infer its total distribution with certainty.

*Haplodrassus omissus*

Although *Haplodrassus omissus* had not been recorded previously in Madeira, it does not come as a surprise to be present there, given its known distribution across the Mediterranean basin and the Canary Islands.

*Haplodrassus* sp. 164

This morphospecies is present in Porto Santo and, possibly, in other locations of the Madeira archipelago. *Haplodrassus* MS 164 belongs to the *dalmatensis* group.

*Haplodrassus* sp. 158

The morphospecies *Haplodrassus* MS158 belongs to a different group from that of *Haplodrassus* MS164, as its genitalia is more similar to species such as *H. deserticola*, which is present in the Canary Islands or *H. minor*, which has been recorded from Europe to Turkey, according to the World Spider Catalogue. It is possible that more species of this group are present in the Madeira archipelago.

*Macarophaeus cultior*

*Macarophaeus cultior* may be a single-island endemic from Madeira. Although the World Spider Catalog and the Banco de Datos de Biodiversidad de Canarias (based on Oromí et al. (2002)) shows records from the Canary Islands (Oromí et al. (2002), according to Wunderlich (Wunderlich 2011), there are no new records or mentions of *M. cultior* in this archipelago.

*Zelotes aeneus*

We classified this species as introduced, because of its distribution throughout Europe up to Azerbaijan.

*Agyneta canariensis*

We have considered this species as native non-endemic, because it is present in the Canary Islands, Selvagens and Madeira.

*Canariellanum* sp. 21

We considered this species as a new endemic species of a genus so far restricted to the Canary Islands.

*Ceratinopsis* spp.

Given the morphological mismatch of this morphospecies with other described species from the region, we have considered it as endemic to the island of Madeira.

*Tenuiphantes* sp. 259

This morphospecies is probably a new *Tenuiphantes* from the archipelago of Madeira and we, therefore, considered it endemic.

*Tenuiphantes tenebricoloides*

This species has been found and cited several times from the forests of Madeira island. There is a citation from the Canary Islands by Denis (Denis 1941) that was based on a single female specimen that has not been revised since then. Furthermore, the species has not been cited again from the Canary Islands and, according to J. Wunderlich – who described a very similar species, *T. canariensis*, from the Canary Islands – Denis probably misidentified the species, given the resemblance in the female epigyne between *T. tenebricoloides* and *T. canariensis*, Therefore, we considered *T. tenebricoloides* as a Madeiran endemic (Wunderlich 1987). Future molecular phylogenetic work might resolve the relationship between these two species.

Mesiotelus cf. grancanariensis

The species *Mesiotelus grancanariensis* has been cited from the south west coast of Portugal and across the entire archipelago of Madeira – in treeless dry habitats and along the central mountain chain of Madeira. Hence the classification of this species as a native species.

Oonops cf. pulcher

Although it is possible that this species is an undescribed cryptic species native to Madeira, based on morphological characters, it is very likely to be *Oonops pulcher* and, therefore, introduced.

*Orchestina* sp. 160

We classified this morphospecies as archipelago-endemic, based on previous findings by L. Crespo in areas with dry habitats of Porto Santo and Desertas.

*Oxyopes* sp. 80

Since this morphospecies has not been described or cited before, it is very likely endemic to the Madeiran archipelago.

*Philodromus* sp. 266

This morphospecies has not been described or cited before, hence our classification as endemic.

Macaroeris cf. desertensis , Macaroeris cf. diligens and *Macaroeris* sp. 8

We considered Macaroeris cf. desertensis , Macaroeris cf. diligens and *Macaroeris* sp. 8 as most likely new undescribed species and, therefore, endemic to the Madeiran archipelago.

*Lasaeola* sp. 268

Based on the morphological features of this morphospecies, we considered it as a new species and, therefore, an archipelago endemic.

*Rhomphaea nasica*

*Rhomphaea nasica* is known to be present in Africa and on the remote island of St. Helena. Specimens of this species have also been identified in modified habitats such as the Funchal Botanical Garden (Madeira Island). Therefore, we considered this species as introduced.

*Theridion hannoniae*

This species has a Mediterranean distribution and, in the Madeiran archipelago, it is usually found in dry, relatively disturbed areas. We classified this species as of *unknown* biogeographic category, given the impossibility of knowing whether it arrived at the Madeiran archipelago naturally or with human intervention.

*Theridion* sp. 89

This morphospecies is most likely a new species, so we considered it to be single-island endemic, probably from laurel forest.

Misumena cf. nigromaculata

Here we considered this morphospecies to be a new endemic species given the little information on *Misumena nigromaculata*, whose female has been described only once and whose male is unknown.

*Xysticus nubilus*

*Xysticus nubilus* has a Mediterranean distribution and is usually found in dry habitats in the Madeiran archipelago. Therefore, as with *Theridion hannoniae*, we classified it as of *unknown* biogeographic category in Madeira.

*Hyptiotes flavidus*

Although the distribution of this species is largely Mediterranean, given that it is usually found in laurel forests, we classified it as native to Madeira.

## Figures and Tables

**Figure 1a.**
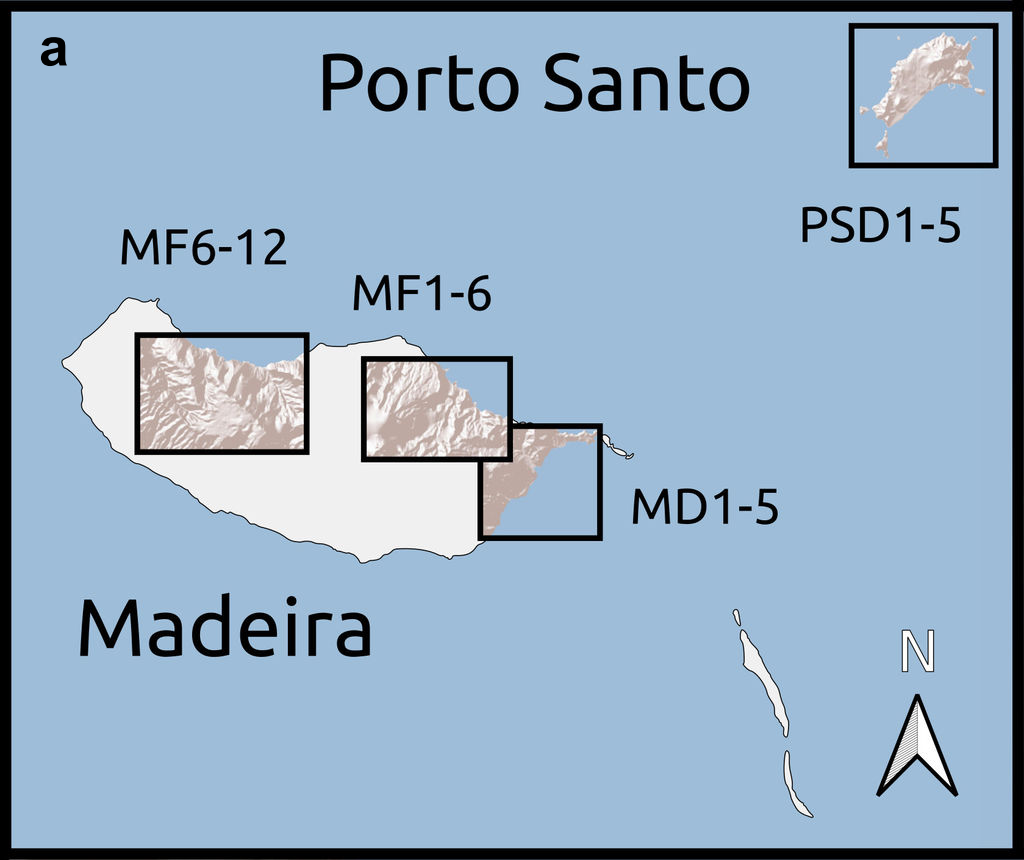
Madeira and Porto Santo Islands

**Figure 1b.**
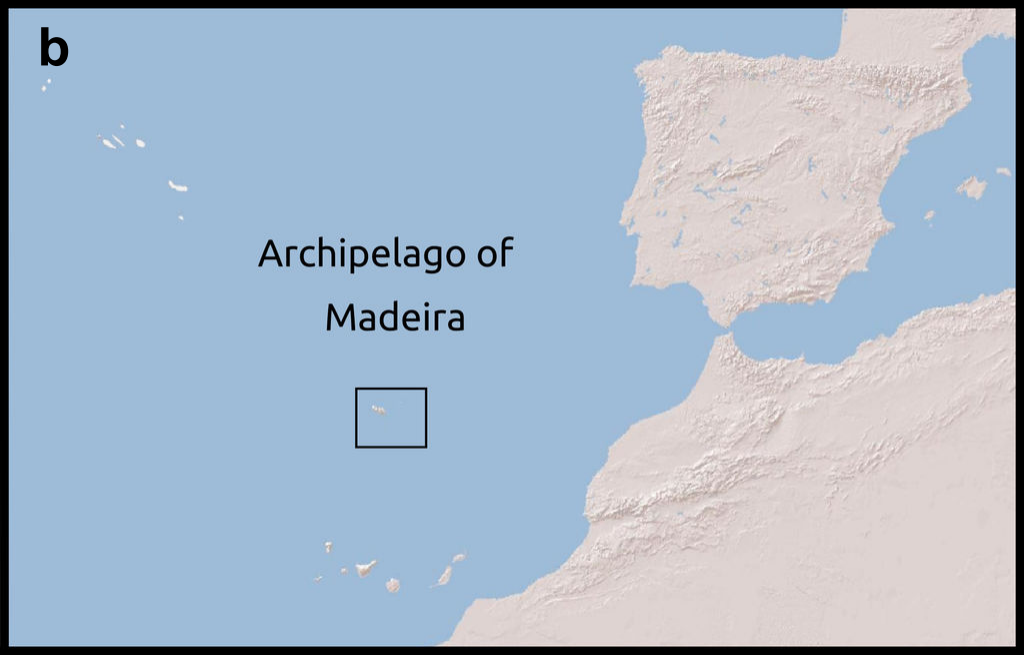
Archipelago of Madeira

**Figure 1c.**
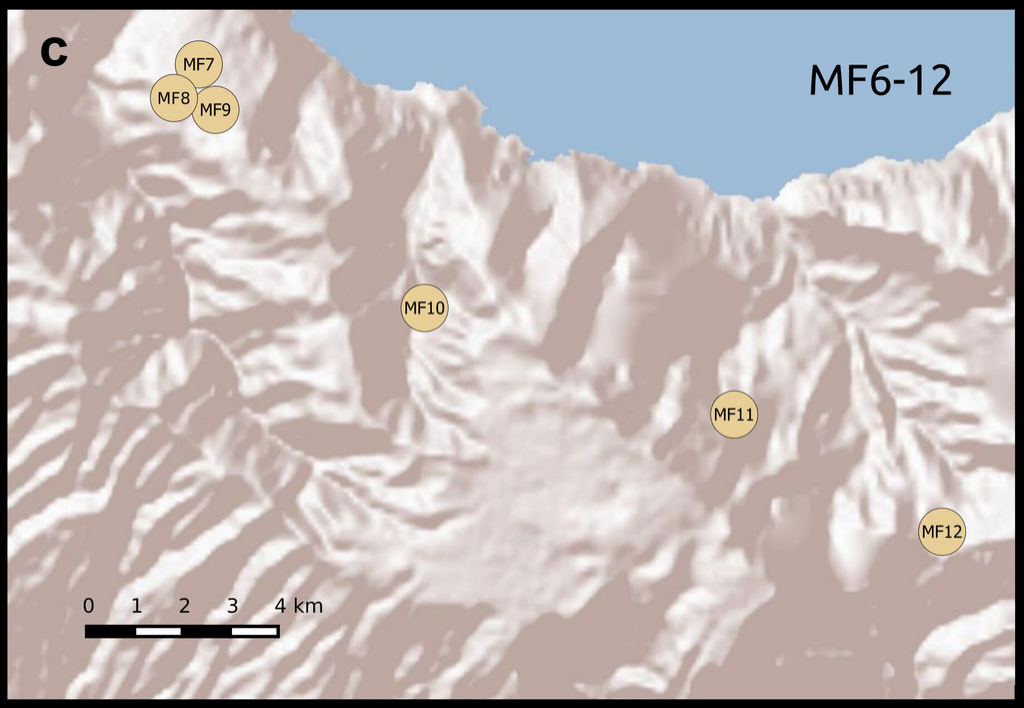
Madeira forest habitat plots 6-12

**Figure 1d.**
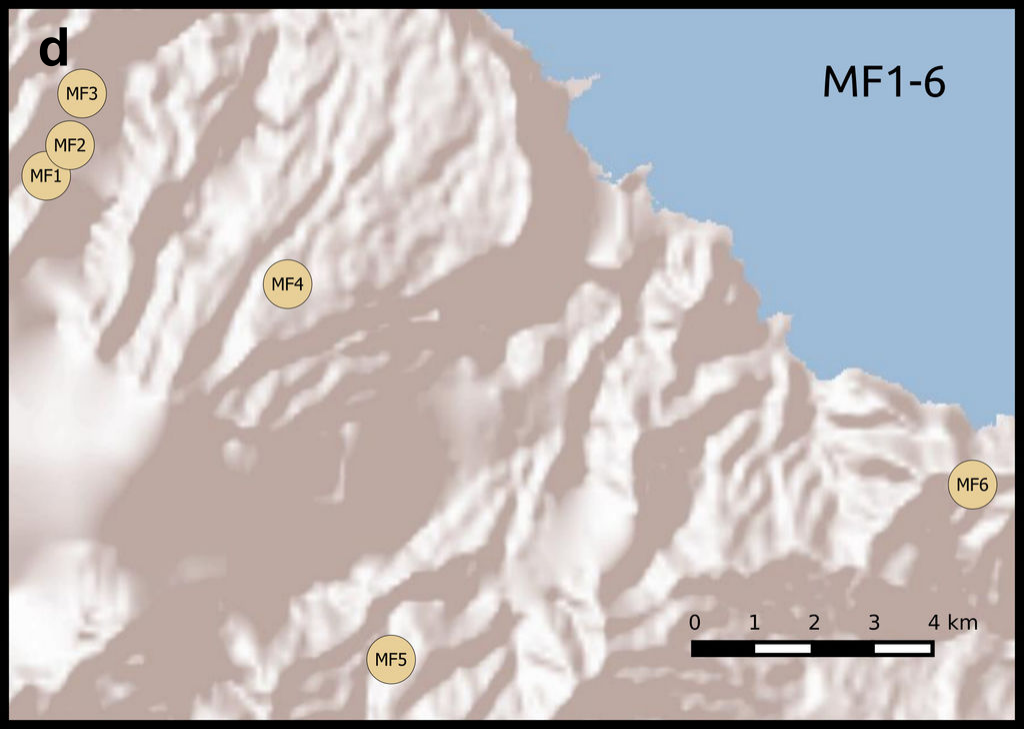
Madeira forest habitat plots 1-6

**Figure 1e.**
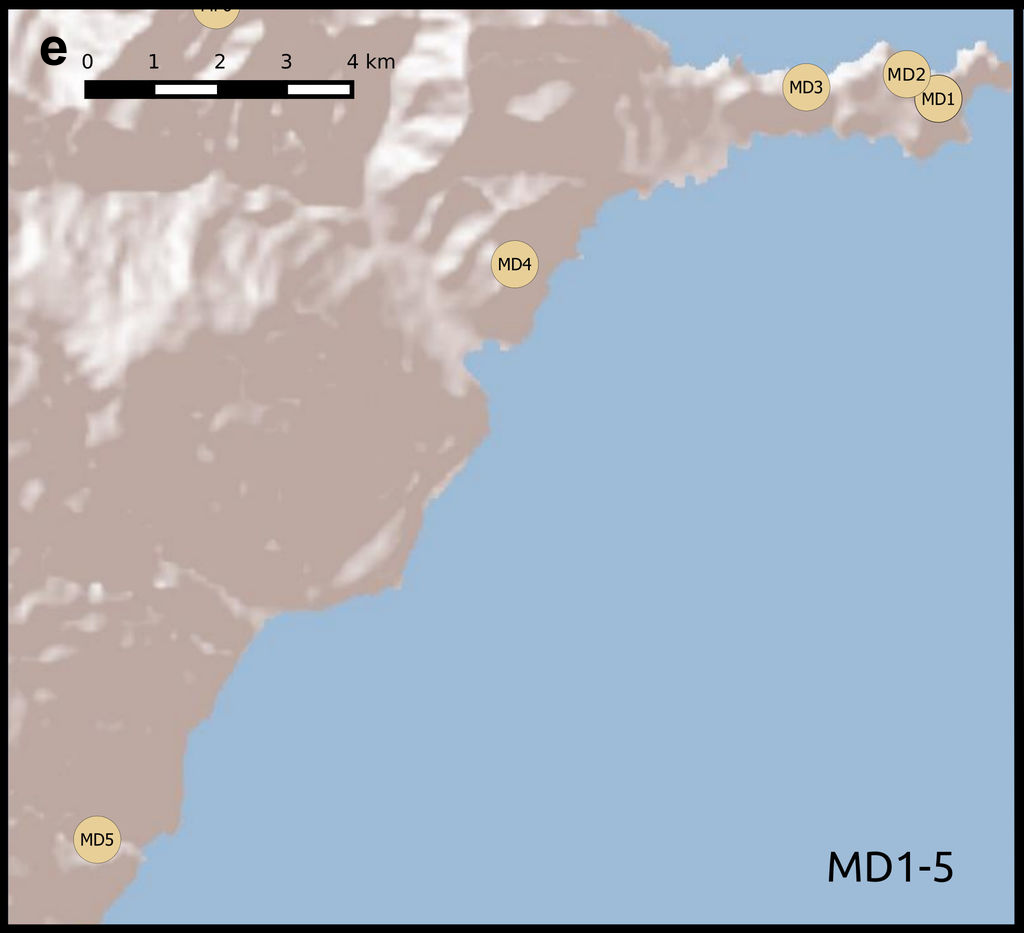
Madeira dry habitat plots 1-5

**Figure 1f.**
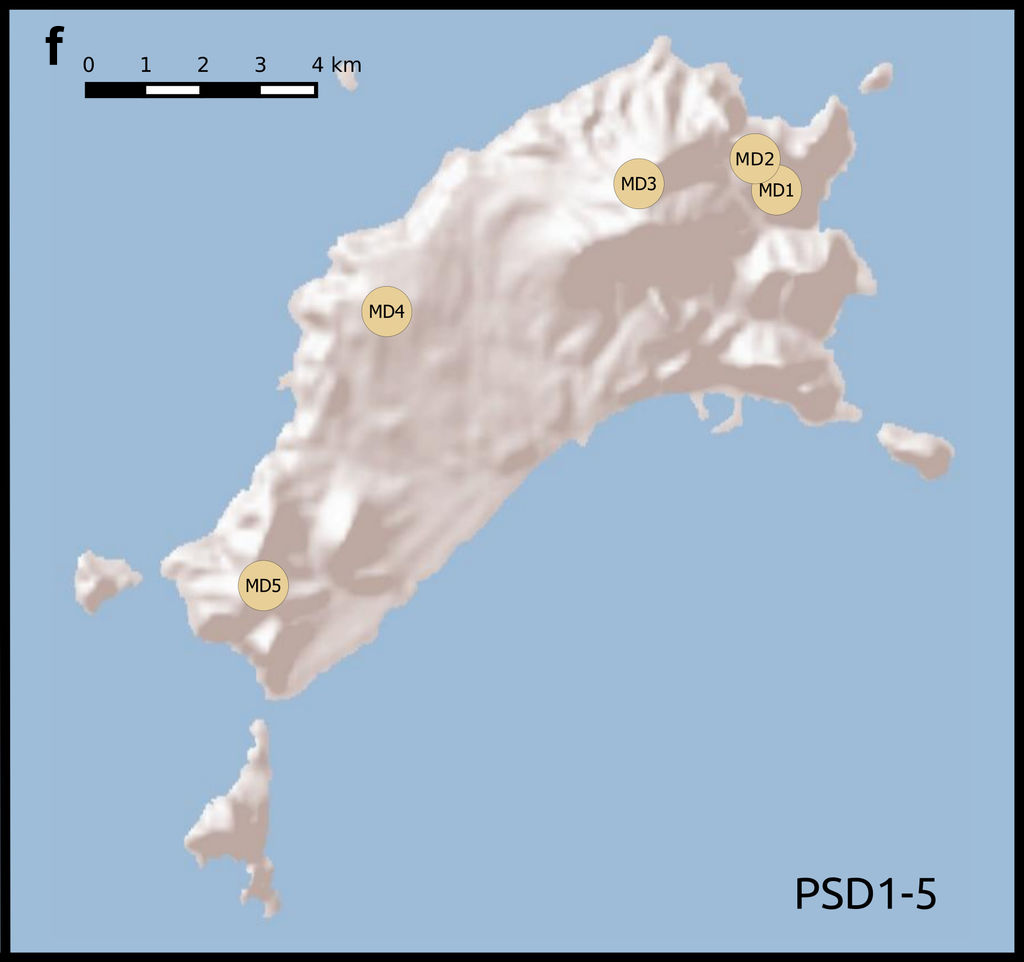
Porto Santo dry habitat plots 1-5

**Table 1.** Coordinates and habitat type of sampling plots.

Plot name (island)	Habitat	Longitude	Latitude
Madeira d1	Dry	-16.70261	32.74633
Madeira d2	Dry	-16.70307	32.74691
Madeira d3	Dry	-16.71886	32.74618
Madeira d4	Dry	-16.75788	32.72623
Madeira d5	Dry	-16.8138	32.66138
Madeira f1	Forest	-16.9371	32.79582
Madeira f2	Forest	-16.93654	32.79801
Madeira f3	Forest	-16.9347	32.80462
Madeira f4	Forest	-16.90233	32.78108
Madeira f5	Forest	-16.88654	32.73302
Madeira f6	Forest	-17.15781	32.82816
Madeira f7	Forest	-17.15871	32.82706
Madeira f8	Forest	-17.15336	32.82306
Madeira f9	Forest	-17.11369	32.79154
Madeira f10	Forest	-17.05499	32.7746
Porto_Santo 1	Dry	-16.30453	33.09135
Porto_Santo 2	Dry	-16.30536	33.0921
Porto_Santo 3	Dry	-16.32426	33.09041
Porto_Santo 4	Dry	-16.36452	33.07376
Porto_Santo 5	Dry	-16.38267	33.03804

**Table 2.** Abundance, biogeographic category and previous records of (morpho)species in six of the forest plots on Madeira island. Abbreviations: Plot names: Madeira forest (MF); Madeira dry habitat (MD); Porto Santo dry habitat (PSD). Biogeographic category (Biog. cat): Endemic (END); Introduced (INT); Macaronesian (MAC); Native non-endemic (NAT); Unknown (UK). Previous records (Prev. Rec.): Madeira (M), Porto Santo (PS), Not recorded (No). Reference for previous records: List of Madeiran Fauna (LMF) (Borges et al. 2008), World Spider Catalogue (WSC) (Natural History Museum Bern 2019).

Family	Species	Biog.Cat.	Prev. Rec.	MF1	MF2	MF3	MF4	MF5	MF6
Agelenidae	*Eratigena feminea* (Simon, 1870)	UK	No	0	0	0	0	0	0
Agelenidae	*Synaphris saphrynis* Lopardo, Hormiga & Melic, 2007	UK	No	0	0	0	0	0	0
Agelenidae	*Tegenaria domestica* (Clerck, 1757)	I	M	0	0	1	0	0	1
Araneidae	*Agalenatea redii* (Scopoli, 1763)	I	M, PS	0	0	0	0	0	0
Araneidae	*Araniella maderiana* (Kulczyński, 1905)	N	M, PS	0	0	0	2	0	0
Araneidae	*Argiope trifasciata* (Forskål, 1775)	UK	M, PS	0	0	0	0	0	0
Araneidae	*Cyclosa maderiana* Kulczyński, 1899	N	M	0	0	0	0	1	0
Araneidae	*Cyrtophora citricola* (Forskål, 1775)	I	M	0	0	0	0	0	0
Araneidae	*Mangora acalypha* (Walckenaer, 1802)	I	M	0	0	0	0	0	0
Araneidae	*Neoscona crucifera* (Lucas, 1838)	I	M, PS	1	1	5	3	7	2
Araneidae	*Zygiella x-notata* (Clerck, 1757)	I	M, PS	0	0	0	0	0	0
Cheiracanthiidae	*Cheiracanthium albidulum* (Blackwall, 1859)	E	M, PS	15	5	28	2	8	12
Clubionidae	*Porrhoclubiona decora* (Blackwall, 1859)	N	M, PS	1	0	0	2	0	3
Dictynidae	*Lathys affinis* (Blackwall, 1862)	E	M, PS	0	1	8	0	11	0
Dictynidae	*Nigma puella* (Simon, 1870)	UK	M, PS	0	0	0	0	0	0
Dysderidae	*Dysdera coiffaiti* Denis, 1962	E	M	0	1	1	0	0	0
Dysderidae	*Dysdera crocata* C.L.Koch, 1838	I	M, PS	0	0	0	0	0	0
Gnaphosidae	*Drassodes lutescens* (C.L.Koch, 1839)	UK	M	0	0	0	0	0	0
Gnaphosidae	*Haplodrassus* sp. 158	E	No	0	0	0	0	0	0
Gnaphosidae	*Haplodrassus* sp. 164	UK	No	0	0	0	0	0	0
Gnaphosidae	*Haplodrassus omissus* (O. Pickard-Cambridge, 1872)	N	No	0	0	0	0	0	0
Gnaphosidae	*Heser hispanus* Senglet, 2012	UK	No	0	0	0	0	0	0
Gnaphosidae	*Macarophaeus cultior* (Kulczyński, 1899)	E	No	0	0	2	0	0	0
Gnaphosidae	*Micaria pallipes* (Lucas, 1846)	UK	M	0	0	0	0	0	0
Gnaphosidae	*Setaphis carmeli* (O.P.-Cambridge, 1872)	UK	No	0	0	0	0	0	0
Gnaphosidae	*Trachyzelotes holosericeus* (Simon, 1878)	UK	M	0	0	0	0	0	0
Gnaphosidae	*Trachyzelotes lyonneti* (Audouin, 1826)	UK	M, PS	0	0	0	0	0	0
Gnaphosidae	*Zelotes aeneus* (Simon, 1878)	I	No (LMF), M (WSC)	0	0	0	0	0	0
Gnaphosidae	*Zelotes tenuis* (L.Koch, 1866)	I	No	0	0	0	0	0	0
Gnaphosidae	*Zimirina lepida* (Blackwall, 1859)	E	M	0	0	0	0	0	0
Linyphiidae	*Agyneta canariensis* Wunderlich, 1987	N	No (LMF), M (WSC)	0	0	0	0	0	0
Linyphiidae	*Agyneta fuscipalpa* (C.L.Koch, 1836)	I	No	0	0	0	0	0	0
Linyphiidae	*Canariellanum* sp. 21	E	No	0	0	0	0	0	10
Linyphiidae	*Centromerus variegatus* Denis, 1962	E	M	0	0	0	2	0	0
Linyphiidae	*Ceratinopsis* sp. 111	E	No	0	0	0	0	0	0
Linyphiidae	*Ceratinopsis acripes* (Denis, 1962)	N	M	3	1	2	2	4	2
Linyphiidae	*Ceratinopsis infuscata* (Denis, 1962)	E	M	0	0	0	1	2	0
Linyphiidae	*Ceratinopsis* sp. 233	E	No	0	0	1	0	0	0
Linyphiidae	*Ceratinopsis* sp. 58	E	No	0	0	1	0	0	0
Linyphiidae	*Diplocephalus graecus* (O.P.-Cambridge, 1873)	I	No	0	0	0	0	0	0
Linyphiidae	*Entelecara schmitzi* Kulczyński, 1905	UK	M	2	5	5	0	5	0
Linyphiidae	*Frontinellina dearmata* (Kulczyński, 1899)	E	M	6	4	3	7	62	0
Linyphiidae	*Frontiphantes fulgurenotatus* (Schenkel, 1938)	E	M	0	0	1	0	3	4
Linyphiidae	*Lepthyphantes mauli* Wunderlich, 1992	E	M	0	0	1	0	0	0
Linyphiidae	*Microctenonyx subitaneus* (O.P.-Cambridge, 1875)	UK	M	0	0	0	0	0	0
Linyphiidae	*Microlinyphia johnsoni* (Blackwall, 1859)	N	M, PS	3	6	7	12	21	8
Linyphiidae	*Ostearius melanopygius* (O.P.-Cambridge, 1880)	I	M	0	0	0	0	0	0
Linyphiidae	*Palliduphantes schmitzi* (Kulczyński, 1899)	N	M	5	0	1	10	0	17
Linyphiidae	*Parapelecopsis nemoralioides* (O. Pickard-Cambridge, 1884)	I	No	0	0	0	0	0	0
Linyphiidae	*Pelecopsis inedita* (O.P.-Cambridge, 1875)	I	No	0	0	0	0	0	0
Linyphiidae	*Tenuiphantes* sp. 259	E	No	0	0	0	0	0	0
Linyphiidae	*Tenuiphantes tenebricoloides* (Schenkel, 1938)	E	M	0	0	0	1	1	0
Linyphiidae	*Tenuiphantes tenuis* (Blackwall, 1852)	I	M, PS	50	35	53	43	34	66
Linyphiidae	*Turinyphia maderiana* (Schenkel, 1938)	E	M	0	0	0	0	5	1
Liocranidae	Mesiotelus cf. grancanariensis Wunderlich, 1992	N	No	0	0	0	0	0	0
Lycosidae	*Hogna insularum* (Kulczyński, 1899)	E	M, PS	0	0	0	0	0	0
Lycosidae	*Hogna schmitzi* Wunderlich, 1992	E	PS	0	0	0	0	0	0
Lycosidae	*Pardosa proxima* (C.L.Koch, 1847)	N	M, PS	0	0	0	0	0	0
Mimetidae	*Ero aphana* (Walckenaer, 1802)	N	M, PS	0	0	1	0	0	1
Mysmenidae	*Trogloneta madeirensis* Wunderlich, 1987	E	M	0	2	3	4	1	13
Nesticidae	*Eidmannella pallida* (Emerton, 1875)	I	M	0	0	0	0	0	0
Oecobiidae	*Oecobius similis* Kulczyński, 1909	N	M, PS	0	0	0	0	0	0
Oonopidae	*Gamasomorpha insularis* Simon, 1907	UK	M	0	0	0	0	0	0
Oonopidae	Oonops cf. pulcher Templeton, 1835	UK	No	0	0	0	0	0	0
Oonopidae	*Opopaea concolor* (Blackwall, 1859)	N	M	0	0	0	0	0	0
Oonopidae	*Orchestina* sp. 160	E	No	0	0	0	0	0	0
Oxyopidae	*Oxyopes* sp. 80	UK	No	0	0	0	0	0	0
Philodromidae	*Philodromus insulanus* Kulczyński, 1905	E	M	0	0	0	0	0	0
Philodromidae	*Philodromus* MAD266	UK	No	0	0	0	0	0	0
Philodromidae	*Thanatus vulgaris* Simon, 1870	UK	M, PS	0	0	0	0	0	0
Pholcidae	*Pholcus madeirensis* Wunderlich, 1987	E	M	3	1	7	0	0	0
Salticidae	*Chalcoscirtus sublestus* (Blackwall, 1867)	N	M	0	0	0	0	0	0
Salticidae	Macaroeris cf. desertensis Wunderlich, 1992	E	No	0	0	0	0	0	0
Salticidae	Macaroeris cf. diligens (Blackwall, 1867)	E	No	0	0	0	0	0	0
Salticidae	*Macaroeris diligens* (Blackwall, 1867)	N	M, PS	3	0	1	1	2	0
Salticidae	*Macaroeris* sp. 8	E	No	0	0	0	0	0	0
Salticidae	*Pellenes maderianus* Kulczyński, 1905	N	M	0	0	0	0	0	0
Scytodidae	*Scytodes velutina* Heineken & Lowe, 1832	UK	M	0	0	0	0	0	0
Segestriidae	*Ariadna maderiana* Warburton, 1892	E	M, PS	0	0	0	0	0	0
Segestriidae	*Segestria florentina* (Rossi, 1790)	I	M, PS	0	0	0	0	0	0
Tetragnathidae	*Meta stridulans* Wunderlich, 1987	E	M	8	7	9	1	2	15
Theridiidae	*Cryptachaea blattea* (Urquhart, 1886)	I	No	3	0	13	9	3	0
Theridiidae	*Dipoenata longitarsis* (Denis, 1962)	E	M	0	0	0	1	0	0
Theridiidae	*Echinotheridion gibberosum* (Kulczyński, 1899)	N	M	37	28	63	4	32	7
Theridiidae	*Enoplognatha diversa* (Blackwall, 1859)	I	M, PS	0	0	0	0	0	0
Theridiidae	*Enoplognatha sattleri* Bösenberg, 1895	N	M	0	0	0	0	1	0
Theridiidae	*Episinus maderianus* Kulczyński, 1905	N	M	182	59	160	42	33	84
Theridiidae	*Kochiura aulica* (C.L.Koch, 1838)	N	M, PS	0	0	0	0	0	0
Theridiidae	*Laseola* sp. 268	E	No	0	0	0	0	0	0
Theridiidae	*Macaridion barreti* (Kulczyński, 1899)	N	M	55	63	112	1	60	15
Theridiidae	*Paidiscura orotavensis* (Schmidt, 1968)	N	M	0	13	1	2	0	0
Theridiidae	*Rhomphaea nasica* (Simon, 1873)	I	M	0	1	2	0	0	1
Theridiidae	*Rugathodes madeirensis* Wunderlich, 1987	E	M	48	2	2	156	8	96
Theridiidae	*Steatoda grossa* (C.L.Koch, 1838)	I	M, PS	0	0	0	0	0	0
Theridiidae	*Steatoda nobilis* (Thorell, 1875)	N	M, PS	1	1	4	0	1	0
Theridiidae	*Theridion hannoniae* Denis, 1945	UK	M	0	0	0	0	0	0
Theridiidae	*Theridion musivivum* Schmidt, 1956	N	M, PS	0	0	0	0	0	0
Theridiidae	*Theridion* sp. 89	E	No	4	2	2	2	11	1
Thomisidae	Misumena cf. nigromaculata Denis, 1963	E	No	0	0	0	0	0	0
Thomisidae	*Misumena spinifera* (Blackwall, 1862)	N	M, PS	1	1	2	0	0	1
Thomisidae	*Thomisus onustus* Walckenaer, 1805	I	No	0	0	0	0	0	0
Thomisidae	*Xysticus nubilus* Simon, 1875	UK	M, PS	0	0	0	0	0	0
Uloboridae	*Hyptiotes flavidus* (Blackwall, 1862)	N	M	13	3	3	11	40	0
Uloboridae	*Uloborus walckenaerius* Latreille, 1806	UK	M	0	0	0	0	0	0
Zodariidae	*Zodarion styliferum* (Simon, 1870)	UK	M	0	0	0	0	0	0
Species richness				21	22	32	24	25	21

**Table 3.** Abundance, biogeographic category and previous records of (morpho)species in five of the forest plots and two of the dry habitat plots on Madeira island. Abbreviations: Madeira forest plot (MF), Madeira dry plot (MD).

Family	Species	MF7	MF8	MF9	MF10	MF11	MF12	MD1	MD2
Agelenidae	*Eratigena feminea* (Simon, 1870)	0	0	0	0	0	0	0	0
Agelenidae	*Synaphris saphrynis* Lopardo, Hormiga & Melic 2007	0	0	0	0	0	0	0	0
Agelenidae	*Tegenaria domestica* (Clerck, 1757)	0	0	0	0	0	0	0	0
Araneidae	*Agalenatea redii* (Scopoli, 1763)	0	0	0	0	0	0	7	22
Araneidae	*Araniella maderiana* (Kulczyński, 1905)	3	0	3	0	0	1	0	0
Araneidae	*Argiope trifasciata* (Forskål, 1775)	0	0	0	0	0	0	10	17
Araneidae	*Cyclosa maderiana* Kulczyński, 1899	0	1	0	0	0	1	0	0
Araneidae	*Cyrtophora citricola* (Forskål, 1775)	0	0	0	0	0	0	0	0
Araneidae	*Mangora acalypha* (Walckenaer, 1802)	0	0	0	0	0	0	2	0
Araneidae	*Neoscona crucifera* (Lucas, 1838)	2	1	1	1	1	2	0	0
Araneidae	*Zygiella x-notata* (Clerck, 1757)	0	0	0	0	0	0	0	0
Cheiracanthiidae	*Cheiracanthium albidulum* (Blackwall, 1859)	34	11	36	9	15	43	0	0
Clubionidae	*Porrhoclubiona decora* (Blackwall, 1859)	2	0	3	2	0	1	1	2
Dictynidae	*Lathys affinis* (Blackwall, 1862)	6	1	2	2	0	3	0	0
Dictynidae	*Nigma puella* (Simon, 1870)	0	0	0	0	0	0	0	0
Dysderidae	*Dysdera coiffaiti* Denis, 1962	3	1	2	0	0	0	0	0
Dysderidae	*Dysdera crocata* C.L.Koch, 1838	0	0	0	0	0	0	2	5
Gnaphosidae	*Drassodes lutescens* (C.L.Koch, 1839)	0	0	0	0	0	0	0	0
Gnaphosidae	*Haplodrassus* sp. 158	0	0	0	0	0	0	0	0
Gnaphosidae	*Haplodrassus* sp. 164	0	0	0	0	0	0	0	0
Gnaphosidae	*Haplodrassus omissus* (O. Pickard-Cambridge, 1872)	0	0	0	0	0	0	13	1
Gnaphosidae	*Heser hispanus* Senglet, 2012	0	0	0	0	0	0	35	23
Gnaphosidae	*Macarophaeus cultior* (Kulczyński, 1899)	0	0	0	0	0	0	0	0
Gnaphosidae	*Micaria pallipes* (Lucas, 1846)	0	0	0	0	0	0	0	0
Gnaphosidae	*Setaphis carmeli* (O.P.-Cambridge, 1872)	0	0	0	0	0	0	0	0
Gnaphosidae	*Trachyzelotes holosericeus* (Simon, 1878)	0	0	0	0	0	0	0	0
Gnaphosidae	*Trachyzelotes lyonneti* (Audouin, 1826)	0	0	0	0	0	0	4	2
Gnaphosidae	*Zelotes aeneus* (Simon, 1878)	0	0	0	0	0	0	0	0
Gnaphosidae	*Zelotes tenuis* (L.Koch, 1866)	0	0	0	0	0	0	0	0
Gnaphosidae	*Zimirina lepida* (Blackwall, 1859)	0	0	0	0	0	0	3	0
Linyphiidae	*Agyneta canariensis* Wunderlich, 1987	0	0	0	0	0	0	0	0
Linyphiidae	*Agyneta fuscipalpa* (C.L.Koch, 1836)	0	0	0	0	0	0	3	9
Linyphiidae	*Canariellanum* sp. 21	0	0	0	0	0	0	0	0
Linyphiidae	*Centromerus variegatus* Denis, 1962	0	0	0	0	0	0	0	0
Linyphiidae	*Ceratinopsis* sp. 111	0	0	0	0	0	1	0	0
Linyphiidae	*Ceratinopsis acripes* (Denis, 1962)	2	1	1	1	4	6	0	0
Linyphiidae	*Ceratinopsis infuscata* (Denis, 1962)	0	2	8	0	1	0	0	0
Linyphiidae	*Ceratinopsis* sp. 233	0	1	0	0	0	0	0	0
Linyphiidae	*Ceratinopsis* sp. 58	0	0	0	0	0	0	0	0
Linyphiidae	*Diplocephalus graecus* (O.P.-Cambridge, 1873)	0	0	0	0	0	0	103	375
Linyphiidae	*Entelecara schmitzi* Kulczyński, 1905	1	0	2	5	2	7	0	0
Linyphiidae	*Frontinellina dearmata* (Kulczyński, 1899)	7	1	4	8	32	43	0	0
Linyphiidae	*Frontiphantes fulgurenotatus* (Schenkel, 1938)	14	0	1	0	2	1	0	0
Linyphiidae	*Lepthyphantes mauli* Wunderlich, 1992	0	0	0	0	0	0	0	0
Linyphiidae	*Microctenonyx subitaneus* (O.P.-Cambridge, 1875)	0	0	0	0	0	0	0	6
Linyphiidae	*Microlinyphia johnsoni* (Blackwall, 1859)	2	3	4	8	8	27	0	0
Linyphiidae	*Ostearius melanopygius* (O.P.-Cambridge, 1880)	0	0	0	0	0	0	0	0
Linyphiidae	*Palliduphantes schmitzi* (Kulczyński, 1899)	3	0	0	5	1	3	0	0
Linyphiidae	*Parapelecopsis nemoralioides* (O. Pickard-Cambridge, 1884)	0	0	0	0	0	0	2	3
Linyphiidae	*Pelecopsis inedita* (O.P.-Cambridge, 1875)	0	0	0	0	0	0	10	15
Linyphiidae	*Tenuiphantes* sp. 259	0	0	0	0	0	0	0	0
Linyphiidae	*Tenuiphantes tenebricoloides* (Schenkel, 1938)	0	0	0	0	1	1	0	0
Linyphiidae	*Tenuiphantes tenuis* (Blackwall, 1852)	31	58	13	49	25	56	0	4
Linyphiidae	*Turinyphia maderiana* (Schenkel, 1938)	0	2	0	1	4	0	0	0
Liocranidae	Mesiotelus cf. grancanariensis Wunderlich, 1992	0	0	0	0	0	0	1	0
Lycosidae	*Hogna insularum* (Kulczyński, 1899)	0	0	0	0	0	0	60	49
Lycosidae	*Hogna schmitzi* Wunderlich, 1992	0	0	0	0	0	0	0	0
Lycosidae	*Pardosa proxima* (C.L.Koch, 1847)	0	0	3	0	0	0	0	0
Mimetidae	*Ero aphana* (Walckenaer, 1802)	0	0	0	2	0	0	0	0
Mysmenidae	*Trogloneta madeirensis* Wunderlich, 1987	0	0	0	0	2	0	0	0
Nesticidae	*Eidmannella pallida* (Emerton, 1875)	0	0	0	0	0	0	0	2
Oecobiidae	*Oecobius similis* Kulczyński, 1909	0	0	0	0	0	0	8	10
Oonopidae	*Gamasomorpha insularis* Simon, 1907	0	0	0	0	0	0	0	1
Oonopidae	Oonops cf. pulcher Templeton, 1835	0	0	0	0	0	0	0	0
Oonopidae	*Opopaea concolor* (Blackwall, 1859)	0	0	0	0	0	0	0	1
Oonopidae	*Orchestina* sp. 160	0	0	0	0	0	0	0	0
Oxyopidae	*Oxyopes* sp. 80	0	0	0	0	0	0	0	0
Philodromidae	*Philodromus insulanus* Kulczyński, 1905	1	0	0	0	0	0	0	0
Philodromidae	*Philodromus* MAD266	0	0	0	0	0	0	0	0
Philodromidae	*Thanatus vulgaris* Simon, 1870	0	0	0	0	0	0	6	6
Pholcidae	*Pholcus madeirensis* Wunderlich, 1987	0	0	0	0	0	0	0	0
Salticidae	*Chalcoscirtus sublestus* (Blackwall, 1867)	0	0	0	0	0	0	1	1
Salticidae	Macaroeris cf. desertensis Wunderlich, 1992	0	0	0	0	0	0	0	0
Salticidae	Macaroeris cf. diligens (Blackwall, 1867)	0	0	0	0	0	0	0	0
Salticidae	*Macaroeris diligens* (Blackwall, 1867)	4	1	8	5	0	1	0	0
Salticidae	*Macaroeris* sp. 8	0	0	0	0	0	0	5	5
Salticidae	*Pellenes maderianus* Kulczyński, 1905	0	0	0	0	0	0	0	4
Scytodidae	*Scytodes velutina* Heineken & Lowe, 1832	0	0	0	0	0	0	0	0
Segestriidae	*Ariadna maderiana* Warburton, 1892	0	0	0	0	0	0	0	0
Segestriidae	*Segestria florentina* (Rossi, 1790)	0	0	0	0	0	0	0	0
Tetragnathidae	*Meta stridulans* Wunderlich, 1987	3	1	3	10	2	1	0	0
Theridiidae	*Cryptachaea blattea* (Urquhart, 1886)	2	3	5	3	6	3	1	0
* Theridiidae *	*Dipoenata longitarsis* (Denis, 1962)	0	0	0	0	0	0	0	0
Theridiidae	*Echinotheridion gibberosum* (Kulczyński, 1899)	39	3	9	1	22	71	0	0
Theridiidae	*Enoplognatha diversa* (Blackwall, 1859)	0	0	0	0	0	0	0	0
Theridiidae	*Enoplognatha sattleri* Bösenberg, 1895	0	0	0	0	0	0	0	0
Theridiidae	*Episinus maderianus* Kulczyński, 1905	64	14	41	38	18	11	0	0
Theridiidae	*Kochiura aulica* (C.L.Koch, 1838)	0	0	0	0	0	0	0	0
Theridiidae	*Laseola* sp. 268	0	0	0	0	0	0	0	0
Theridiidae	*Macaridion barreti* (Kulczyński, 1899)	14	0	17	19	19	119	0	0
Theridiidae	*Paidiscura orotavensis* (Schmidt, 1968)	3	1	1	15	2	0	0	0
Theridiidae	*Rhomphaea nasica* (Simon, 1873)	0	0	0	0	0	0	0	0
Theridiidae	*Rugathodes madeirensis* Wunderlich, 1987	1	1	0	0	5	6	0	0
Theridiidae	*Steatoda grossa* (C.L.Koch, 1838)	0	0	0	0	0	0	2	4
Theridiidae	*Steatoda nobilis* (Thorell, 1875)	6	7	2	0	2	0	0	0
Theridiidae	*Theridion hannoniae* Denis, 1945	0	0	0	0	0	0	0	0
Theridiidae	*Theridion musivivum* Schmidt, 1956	0	0	0	1	0	0	0	0
Theridiidae	*Theridion* sp. 89	4	0	0	2	0	0	0	0
Thomisidae	Misumena cf. nigromaculata Denis, 1963	0	0	0	0	0	0	0	1
Thomisidae	*Misumena spinifera* (Blackwall, 1862)	1	0	4	0	0	2	0	0
Thomisidae	*Thomisus onustus* Walckenaer, 1805	0	0	0	0	0	0	0	0
Thomisidae	*Xysticus nubilus* Simon, 1875	0	0	0	0	0	0	9	2
Uloboridae	*Hyptiotes flavidus* (Blackwall, 1862)	10	1	5	2	15	8	0	0
Uloboridae	*Uloborus walckenaerius* Latreille, 1806	0	0	0	0	0	0	0	1
Zodariidae	*Zodarion styliferum* (Simon, 1870)	0	0	0	0	0	0	0	0
Species richness		26	21	24	22	22	24	22	26

**Table 4.** Abundance of (morpho)species in three of the plots on Madeira island and in the plots on Porto Santo island. Abbreviations: Madeira dry plot (MD), Porto Santo plot (PS).

Family	Species	MD3	MD4	MD5	PSD1	PSD2	PSD3	PSD4	PSD5	Total
Agelenidae	*Eratigena feminea* (Simon, 1870)	0	0	1	0	0	0	0	0	1
Agelenidae	*Synaphris saphrynis* Lopardo, Hormiga & Melic, 2007	0	0	0	0	0	0	0	19	19
Agelenidae	*Tegenaria domestica* (Clerck, 1757)	0	0	0	0	0	0	0	0	2
Araneidae	*Agalenatea redii* (Scopoli, 1763)	2	28	19	1	1	0	0	0	80
Araneidae	*Araniella maderiana* (Kulczyński, 1905)	0	0	0	0	0	0	0	0	9
Araneidae	*Argiope trifasciata* (Forskål, 1775)	1	38	4	0	0	0	0	1	71
Araneidae	*Cyclosa maderiana* Kulczyński, 1899	0	0	0	0	0	0	0	0	3
Araneidae	*Cyrtophora citricola* (Forskål, 1775)	0	0	1	0	0	0	0	0	1
Araneidae	*Mangora acalypha* (Walckenaer, 1802)	1	7	22	1	0	0	0	0	33
Araneidae	*Neoscona crucifera* (Lucas, 1838)	0	0	0	1	0	0	0	0	28
Araneidae	*Zygiella x-notata* (Clerck, 1757)	0	0	1	0	0	0	0	0	1
Cheiracanthiidae	*Cheiracanthium albidulum* (Blackwall, 1859)	0	0	0	0	0	0	0	0	218
Clubionidae	*Porrhoclubiona decora* (Blackwall, 1859)	10	7	3	3	3	0	0	9	52
Dictynidae	*Lathys affinis* (Blackwall, 1862)	0	0	0	16	18	2	0	0	70
Dictynidae	*Nigma puella* (Simon, 1870)	0	0	15	0	0	0	0	0	15
Dysderidae	*Dysdera coiffaiti* Denis, 1962	0	0	0	0	0	0	0	0	8
Dysderidae	*Dysdera crocata* C.L.Koch, 1838	5	8	18	2	0	3	0	0	43
Gnaphosidae	*Drassodes lutescens* (C.L.Koch, 1839)	0	0	0	0	0	0	1	0	1
Gnaphosidae	*Haplodrassus* sp. 158	0	0	0	18	7	0	25	18	68
Gnaphosidae	*Haplodrassus* sp. 164	0	0	0	6	5	0	5	2	18
Gnaphosidae	*Haplodrassus omissus* (O. Pickard-Cambridge, 1872)	6	0	0	0	1	0	13	1	35
Gnaphosidae	*Heser hispanus* Senglet, 2012	1	0	0	0	0	0	0	0	59
Gnaphosidae	*Macarophaeus cultior* (Kulczyński, 1899)	0	0	0	0	0	0	0	0	2
Gnaphosidae	*Micaria pallipes* (Lucas, 1846)	1	0	0	0	0	0	1	1	3
Gnaphosidae	*Setaphis carmeli* (O.P.-Cambridge, 1872)	0	0	0	1	1	6	0	0	8
Gnaphosidae	*Trachyzelotes holosericeus* (Simon, 1878)	1	0	0	7	4	1	0	0	13
Gnaphosidae	*Trachyzelotes lyonneti* (Audouin, 1826)	0	7	1	4	0	17	0	2	37
Gnaphosidae	*Zelotes aeneus* (Simon, 1878)	0	0	0	4	0	0	2	1	7
Gnaphosidae	*Zelotes tenuis* (L.Koch, 1866)	1	0	0	0	0	0	0	0	1
Gnaphosidae	*Zimirina lepida* (Blackwall, 1859)	0	1	0	0	0	1	0	0	5
Linyphiidae	*Agyneta canariensis* Wunderlich, 1987	3	0	0	28	7	3	0	5	46
Linyphiidae	*Agyneta fuscipalpa* (C.L.Koch, 1836)	2	66	15	8	1	23	10	13	150
Linyphiidae	*Canariellanum* sp. 21	0	0	0	0	0	0	0	0	10
Linyphiidae	*Centromerus variegatus* Denis, 1962	0	0	0	0	0	0	0	0	2
Linyphiidae	*Ceratinopsis* sp. 111	0	0	0	0	0	0	0	0	1
Linyphiidae	*Ceratinopsis acripes* (Denis, 1962)	0	0	0	0	0	0	0	0	29
Linyphiidae	*Ceratinopsis infuscata* (Denis, 1962)	0	0	0	0	0	0	0	0	14
Linyphiidae	*Ceratinopsis* sp. 233	0	0	0	0	0	0	0	0	2
Linyphiidae	*Ceratinopsis* sp. 58	0	0	0	0	0	0	0	0	1
Linyphiidae	*Diplocephalus graecus* (O.P.-Cambridge, 1873)	33	2	6	12	3	76	1	9	620
Linyphiidae	*Entelecara schmitzi* Kulczyński, 1905	0	0	0	0	0	0	0	0	34
Linyphiidae	*Frontinellina dearmata* (Kulczyński, 1899)	0	0	0	0	0	0	0	0	177
Linyphiidae	*Frontiphantes fulgurenotatus* (Schenkel, 1938)	0	0	0	0	0	0	0	0	26
Linyphiidae	*Lepthyphantes mauli* Wunderlich, 1992	0	0	0	0	0	0	0	0	1
Linyphiidae	*Microctenonyx subitaneus* (O.P.-Cambridge, 1875)	3	0	0	2	3	8	0	4	26
Linyphiidae	*Microlinyphia johnsoni* (Blackwall, 1859)	0	1	0	0	0	0	0	0	110
Linyphiidae	*Ostearius melanopygius* (O.P.-Cambridge, 1880)	0	0	0	0	0	0	2	0	2
Linyphiidae	*Palliduphantes schmitzi* (Kulczyński, 1899)	0	0	0	0	0	0	0	0	45
Linyphiidae	*Parapelecopsis nemoralioides* (O. Pickard-Cambridge, 1884)	4	0	0	0	0	0	0	0	9
Linyphiidae	*Pelecopsis inedita* (O.P.-Cambridge, 1875)	3	0	0	0	0	0	0	0	28
Linyphiidae	*Tenuiphantes* sp. 259	0	0	0	0	0	1	0	0	1
Linyphiidae	*Tenuiphantes tenebricoloides* (Schenkel, 1938)	0	0	0	0	0	0	0	0	4
Linyphiidae	*Tenuiphantes tenuis* (Blackwall, 1852)	5	0	14	0	1	2	0	1	540
Linyphiidae	*Turinyphia maderiana* (Schenkel, 1938)	0	0	0	0	0	0	0	0	13
Liocranidae	Mesiotelus cf. grancanariensis Wunderlich, 1992	0	0	0	20	5	0	1	2	29
Lycosidae	*Hogna insularum* (Kulczyński, 1899)	54	0	1	6	4	32	34	32	272
Lycosidae	*Hogna schmitzi* Wunderlich, 1992	0	0	0	7	2	0	0	5	14
Lycosidae	*Pardosa proxima* (C.L.Koch, 1847)	0	0	0	0	0	0	0	0	3
Mimetidae	*Ero aphana* (Walckenaer, 1802)	0	0	0	0	0	0	0	0	4
Mysmenidae	*Trogloneta madeirensis* Wunderlich, 1987	0	0	0	0	0	0	0	0	25
Nesticidae	*Eidmannella pallida* (Emerton, 1875)	0	0	0	1	0	0	0	0	3
Oecobiidae	*Oecobius similis* Kulczyński, 1909	82	0	152	5	8	28	1	0	294
Oonopidae	*Gamasomorpha insularis* Simon, 1907	1	0	0	0	0	0	0	0	2
Oonopidae	Oonops cf. pulcher Templeton, 1835	2	0	0	0	0	1	0	0	3
Oonopidae	*Opopaea concolor* (Blackwall, 1859)	0	2	0	0	0	0	0	0	3
Oonopidae	*Orchestina* sp. 160	0	0	0	2	0	0	0	0	2
Oxyopidae	*Oxyopes* sp. 80	0	0	2	0	0	0	0	0	2
Philodromidae	*Philodromus insulanus* Kulczyński, 1905	0	0	0	0	0	0	0	0	1
Philodromidae	*Philodromus* MAD266	0	0	0	0	0	0	1	1	2
Philodromidae	*Thanatus vulgaris* Simon, 1870	10	5	0	2	8	0	8	4	49
Pholcidae	*Pholcus madeirensis* Wunderlich, 1987	0	0	0	0	0	0	0	0	11
Salticidae	*Chalcoscirtus sublestus* (Blackwall, 1867)	2	1	20	3	3	1	1	1	34
Salticidae	Macaroeris cf. desertensis Wunderlich, 1992	0	0	0	7	1	0	2	7	17
Salticidae	Macaroeris cf. diligens (Blackwall, 1867)	0	0	0	3	7	5	1	26	42
Salticidae	*Macaroeris diligens* (Blackwall, 1867)	0	0	0	0	0	0	0	0	26
Salticidae	*Macaroeris* sp. 8	8	4	4	1	0	0	0	0	27
Salticidae	*Pellenes maderianus* Kulczyński, 1905	2	0	0	0	0	0	0	0	6
Scytodidae	*Scytodes velutina* Heineken & Lowe, 1832	0	0	3	0	0	0	0	0	3
Segestriidae	*Ariadna maderiana* Warburton, 1892	0	0	0	1	1	0	0	0	2
Segestriidae	*Segestria florentina* (Rossi, 1790)	0	3	6	0	0	1	0	0	10
Tetragnathidae	*Meta stridulans* Wunderlich, 1987	0	0	0	0	0	0	0	0	62
Theridiidae	*Cryptachaea blattea* (Urquhart, 1886)	0	1	0	0	1	0	0	0	53
Theridiidae	*Dipoenata longitarsis* (Denis, 1962)	0	0	0	0	0	0	0	0	1
Theridiidae	*Echinotheridion gibberosum* (Kulczyński, 1899)	0	0	0	0	0	0	0	0	316
Theridiidae	*Enoplognatha diversa* (Blackwall, 1859)	1	0	0	0	1	1	1	0	4
Theridiidae	*Enoplognatha sattleri* Bösenberg, 1895	0	0	0	0	0	0	0	0	1
Theridiidae	*Episinus maderianus* Kulczyński, 1905	0	0	0	0	0	0	0	0	746
Theridiidae	*Kochiura aulica* (C.L.Koch, 1838)	0	1	0	0	0	0	0	0	1
Theridiidae	*Laseola* sp. 268	0	0	0	0	0	0	0	1	1
Theridiidae	*Macaridion barreti* (Kulczyński, 1899)	0	0	0	0	0	0	0	0	494
Theridiidae	*Paidiscura orotavensis* (Schmidt, 1968)	0	0	0	1	2	19	0	17	77
Theridiidae	*Rhomphaea nasica* (Simon, 1873)	0	0	0	0	0	0	0	0	4
Theridiidae	*Rugathodes madeirensis* Wunderlich, 1987	0	0	0	0	0	0	0	0	325
Theridiidae	*Steatoda grossa* (C.L.Koch, 1838)	1	2	4	0	0	0	0	0	13
Theridiidae	*Steatoda nobilis* (Thorell, 1875)	0	0	0	0	0	0	0	0	24
Theridiidae	*Theridion hannoniae* Denis, 1945	0	0	0	1	0	0	0	0	1
Theridiidae	*Theridion musivivum* Schmidt, 1956	0	0	0	0	0	0	0	0	1
Theridiidae	*Theridion* sp. 89	0	0	0	0	0	0	0	0	28
Thomisidae	Misumena cf. nigromaculata Denis, 1963	0	0	0	0	0	0	0	0	1
Thomisidae	*Misumena spinifera* (Blackwall, 1862)	0	0	0	0	0	0	0	0	12
Thomisidae	*Thomisus onustus* Walckenaer, 1805	0	0	0	0	1	1	0	0	2
Thomisidae	*Xysticus nubilus* Simon, 1875	16	38	4	12	10	7	0	3	101
Uloboridae	*Hyptiotes flavidus* (Blackwall, 1862)	0	0	0	0	0	0	0	0	111
Uloboridae	*Uloborus walckenaerius* Latreille, 1806	1	4	7	0	0	0	0	0	13
Zodariidae	*Zodarion styliferum* (Simon, 1870)	16	13	810	0	0	0	2	9	850
Species richness		30	21	24	31	27	22	19	26	7462
